# Health and safety risks affecting part-time nursing students

**DOI:** 10.4102/hsag.v26i0.1404

**Published:** 2021-04-01

**Authors:** Lorato G. Manyeneng, Mogale L. Pilusa, Mmataniele S. Mogotlane

**Affiliations:** 1 Department of Nursing, Faculty of Science, Tshwane University of Technology, Pretoria, South Africa

**Keywords:** health, safety, stress, fatigue, multitasking, workload, personal development

## Abstract

**Background:**

Professional nurses who are employed full-time can study their postgraduate programmes part-time to add to the basic qualification they obtained through relevant institutions of higher education like universities or colleges. Although there are advantages for part-time study such as improvement of qualifications, enhancement of personal development and increased chance of promotion, there are disadvantages as well, which includes keeping a job, attending to family and social responsibilities, time management to ensure that studying is done after work or in-between work and other activities, attending to assignments and complying with the requirements to acquire the qualification.

**Aim:**

This study aimed to explore health and safety risks faced by nurses who work and study part-time.

**Setting:**

The study was conducted at a university in Gauteng province, South Africa. Nurses who undertake part-time studies at this university were the focus of study.

**Method:**

The research design used in the study was an exploratory, quantitative method that was contextual in nature. Data were collected using a self-administered questionnaire that comprised of demographical and health and safety aspects. Data were analysed by means of descriptive statistics using the Statistics package for Social Sciences version 26.

**Results:**

Research findings indicated that students who study part-time experience health and safety risks such as fatigue (*n* = 86; 49%), stress (*n* = 95; 54%), sleep disorders (*n* = 60; 34%), poor eating habits (*n* = 123; 70%), abuse of caffeine (*n* = 91; 52%) and are prone to road accidents (*n* = 54; 31%).

**Conclusion:**

Nurses who work and study part-time need support from their employers and families. Employers should grant study leave as a way of support.

## Introduction

The opportunity to study part-time is essential, as it enables individuals who cannot study full-time to improve their academic and professional qualifications, in many instances whilst keeping their jobs. Employed students have been described by Cozma (2015:1210) as mature persons, above the normal age of traditional schooling, who undertake studying to enhance their employability and to increase their chances of promotion at work (Butcher & Rose-Adams 2015:134). Other reasons, according to Callender and Little (2015:256) could be to position themselves for later career changes or, for those who are ambitious, self-actualisation. Most nurses undertake part-time study to improve their qualifications, enhance personal development and competency, and indeed, enhance employability. In addition to the personal benefits, post-school studies contribute to the growth of the country’s economy through skills development (Callender & Little 2015:256). The nursing profession offers part-time study opportunities to qualifying nurses on many platforms. Professional nurses who are employed full-time can study part-time to add to the basic qualification they obtained through relevant institutions of higher education such as universities or colleges. Institutions on the other hand may meet students halfway by presenting the programme in blocks of weeks throughout the study period where students attend classes for a given period and go back to work in the agreed period for the employers. In this way, students can keep their employment and progress with their studies simultaneously.

Although there are advantages of part-time study, individuals who work and study have a number of challenges. These include keeping a job, attending to family and social responsibilities, managing time to study after work or in-between work and other activities, attending to assignments and complying with the requirements to acquire the qualification (Booth & Schwartz 2012:44). For those students who have no financial support, studying can be very expensive, with the financial burden impacting negatively on the family (Triventi 2014:1). If these challenges are not well managed, they may predispose working students to health and safety risks such as fatigue and stress.

This study was conducted to explore the health and safety risks faced by students who are working and need to acquire an additional certificate in occupational health nursing science.

## Research purpose and objectives

The aim of the study was to determine the occupational health and safety risks faced by part-time students studying towards a post-basic qualification (specifically, a B.Tech in occupational health nursing).

## Research methods and design

The research design used in the study was a descriptive, exploratory, quantitative method that was contextual in nature. This design was used to gather data to explore the health and safety risks affecting professional nurses who were studying part-time towards a B.Tech in occupational health nursing.

### Setting

The study was conducted at a conveniently selected university in Gauteng, South Africa. The university has a nursing department that offers a basic B.Tech nursing programme, a postgraduate B.Tech programme in oncology nursing and a postgraduate B.Tech programme in occupational health nursing. Both the postgraduate programmes are offered on a part-time basis. The department has a total of 375 occupational health nursing students who have to complete five theoretical subjects and a 900-h practical module before they can register with the statutory body as qualified occupational health nurses. The occupational health nursing programme is run on a block system. Students attend classes at the university for face-to-face teaching and learning 1 week per month for 8 months in an academic year. To comply with work-integrated learning, students are allocated in accredited facilities for their work-integrated learning.

### Population

The target population for the study was nurses who were studying part-time at a university. The accessible population was 375 employed professional nurses who were studying part-time towards a B.Tech qualification in occupational health nursing.

### Sample and sampling technique

A non-probability sampling technique was used to conveniently sample 191 respondents for the study as advised by the statistician as this number consisted of 50% of the population and therefore provided the necessary attributes for the study. The first 191 students were sampled, recruited and selected during the block class attendance at the respective university because of their availability. The inclusion criteria for the respondents were that they had to be:

- Registered at the selected university as B.Tech occupational health nursing students in the first or second year of study.
- Employed for more than a year at their place of work.

### Data collection

A self-administered questionnaire with open- and close-ended questions was given to 191 sampled respondents. The questionnaire was self-developed by the researcher with the assistance of the statistician and had three sections. Section A comprised of two demographic data questions. Section B comprised of 10 health risks questions of which five were close-ended and five were open-ended questions. Section C comprised of two safety questions of which one was open-ended and the other was close-ended question. The accuracy of the instrument was valid as it measured what was to be measured.

### Pre-testing of the data collection instrument

The instrument was pre-tested on 10 professional nurses who complied with the inclusion criteria but were not part of the respondents in the main study. Pre-testing was carried out to establish the validity and reliability of the instrument and to:

- Determine the ease with which the respondents understood the questions asked in the instrument.
- Give the researcher and the field workers practice on how to present the questions to the respondents.
- Determine the time it would take to complete the questionnaire which was found to be 30 min.

### Data collection process

All sampled respondents signed an informed consent form before taking part in the study. The self-administered questionnaire was distributed to the first 191 conveniently sampled respondents when they came to class on their block dates, for them to complete and return to the researcher on an agreed-upon date, which was the next block for attendance. This gave respondents a month to complete the questionnaire. A total of 175 (*n* = 92%) questionnaires were used because 11 (*n* = 6%) respondents did not return the questionnaires and five (*n* = 3%) questionnaires were not properly completed and therefore spoiled. Data collection took 2 months from March until May 2018.

### Data analysis

Data were analysed by means of descriptive statistics using version 26 of the Statistics Package for Social Sciences in order to synthesise and describe the collected data.

### Ethical considerations

Ethical clearance was obtained from the the Tshwane University of Technology (FCRE: 2017/08/001[SCI][2]).

Ethical clearance was obtained from the ethical committee of the university where the study was conducted before collection of data. Permission to conduct the study was also obtained from the nursing department where the respondents in the study were registered. Each respondent was requested to sign an informed consent form before data collection could commence. Respondents were assured of confidentiality and anonymity, as information collected would not be linked to any respondent, and respondents’ names would not appear in any document generated from the study. Respondents could withdraw at any stage from the study with no fear of retribution.

## Research results

The results discussed in the below section include demographic information and experiences with regard to the health and safety risks faced by respondents.

### Demographic information

The demographic information (see Table 1) includes the gender of respondents and their marital status. From the 175 respondents who took part in the study, 33 (19%) were males and 142 (81%) were females. A total of 82 (47%) respondents were married, 81 (46%) were single, eight (5%) were divorced whilst four (2%) were widowed.

**TABLE 1:** Demographic findings of the study.

Demographic type	Demographic	*N* = 175	%	Type of question
Gender	Male	33	19	Close-ended question
	Female	142	81	
Marital status	Married	82	47	Close-ended question
	Single	81	46	
	Divorced	8	5	
	Widowed	4	2	

### Health risks

There were five close-ended and five open-ended questions, which were asked to respondents concerning the health risks that they experienced. Respondents indicated different health risks that they perceived to be directly or indirectly associated with both their work activities and part-time studies. For health risks, an open-ended question was asked on what health risks they were exposed to. Respondents indicated the following: fatigue because of multitasking and extended hours of work, stress, sleeping disorders, time management problems, financial problems, consumption of caffeine to try and keep active and consumption of fast foods because of a lack of time to prepare healthy meals. For some respondents, a lack of study leave resulted in issues with class attendance, (see Table 3) which added more stress. Respondents explained that their inability to attend classes reduced their success rate and poor performance would mean money wasted and more stress.

Respondents also mentioned that fatigue would reduce their resistance to infection and lead to a lack of concentration that would result in injuries such as needle-stick injuries and lawsuits for the administration of wrong medicines.

Results from some of the other variables include:

#### Lack of study leave and class attendance

Only 54 (31%) respondents indicated that they were granted study leave, which entailed financial assistance, time off to attend classes, study days before writing examinations and leave on examination days. Ninety-five respondents (55%) reported having financial problems because of not being granted study leave. Of the 121 (100%) respondents who were not granted study leave, 39 (32%) used their off-duty time to attend class, 11 (9%) used faked illness and sick leave, 10 (8%) used their annual leave and five (4%) took unpaid leave to attend classes. The most worrying finding was that 56 (46%) respondents worked during the night and attended classes during the day without resting. The implication of this was a lack of time to rest as off-duty time and annual leave were used to attend classes, resulting in fatigue.

#### Time management

A total of 111 (63%) respondents indicated an inability to balance work, study and social time, resulting in fatigue. In an effort to manage time, 111 (63%) respondents worked in shifts that included night duty. These shifts exposed them to long and irregular working hours, which resulted in fatigue.

#### Sleeping disorders

A total of 60 (34%) respondents indicated that they had sleeping disorders because they studied whilst working. As a result, 16 (27%) respondents used sleeping tablets to induce sleep, six (10%) took antihistamine treatments that led to drowsiness and made them sleepy, five (8%) took caffeine to counteract sleep and one (2%) abused alcohol. A total of 32 (53%) respondents indicated that they did nothing to address their sleeping disorders.

#### Consumption of caffeine

Some respondents (*n* = 91; 52%) consumed coffee and/or energy drinks. From the 91 (100%) that consumed caffeine or energy drinks, the reasons given were that 57 (63%) consumed caffeine or energy drinks to be alert as they were exhausted most of the time, 13 (14%) did so to remain awake when they felt sleepy and 10 (11%) did so for pleasure. A further 11 (12%) respondents did not give any reason for consuming caffeine or energy drinks (see Table 2). According to Turnbull et al. (2017:169–175), caffeine consumption predisposes the consumer’s total cardiovascular system to coronary heart diseases, arrhythmias, heart failure, cardiac arrest, stroke and hypertension.

**TABLE 2:** Health findings of the study.

Variable	*N*	%	Type of question
**Health risks**			
Fatigue	86	49	Open-ended question
Sleeping disorders	60	34	Open-ended question
Consumption of caffeine	91	52	Both close-ended and open-ended question
Consumption of fast food	122	70	Both close-ended and open-ended question
Stress	95	54	Open-ended question
**Socio-economic problems contributing to health risks**			
No study leave	121	69	Both close-ended and open-ended question
Had study leave	54	31	Both close-ended and open-ended question
Had financial problems	95	55	Both close-ended and open-ended question
Had no Financial problems	80	45	Both close-ended and open-ended question
Had time management problems	111	64	Both close-ended and open-ended question

Note: *N* = 175.

**TABLE 3:** Safety risk findings of the study.

Safety risks	*N*	%	Type of question
**Travelling distance (km)**			
0–30	0	0	Open-ended questions
31–60	36	31	
61–100	24	14	
101–150	16	9	
150 and more	54	31	
Fatigue	54	31	Open-ended question
Physical abuse	26	19	Open-ended question

Note: *N* = 175.

#### Consumption of fast food

A total of 122 (70%) respondents acknowledged that they consumed fast food as they did not have time to prepare food. The health risk of fast food is that it is high in calories, carbohydrates and sugar. Therefore, consuming fast food can result in obesity.

#### Fatigue and stress

As shown in Table 2, of the 175 respondents who took part in the study, 86 (*n* = 49%) indicated that studying whilst working resulted in fatigue, whilst 95 (*n* = 54%) reported stress.

### Safety risks

Two questions were asked on safety risks. A close-ended question was asked on the distance travelled by respondents from their institution of learning, whilst an open-ended question was asked on their safety risks associated with their part time study whilst working. Fifty-four (*n* = 31%) respondents indicated that they travelled more than 150 km to their place of study. Travelling is tiring in itself, and in this study, it contributed to the fatigue experienced. The lack of concentration because of fatigue increases the risk of accidents. Twenty-six (*n* = 19%) respon dents indicated that they were abused physically and emotionally by partners who felt neglected and frustrated by the absence or lack of attention exhibited by the students.

## Discussion of findings

The objective of this study was to explore the health risks experienced by employed professional nurse students studying towards an academic qualification, occupational health nursing. There were advantages for studying whilst working even though health and safety were in some instances compromised. The study results have confirmed the stipulation by Polidano and Zakirova (2011:11) when they stated that studying and working has positive and negative outcomes, for example, promotions, fatigue and stress.

### Health risks

#### Fatigue and stress

It was clear from the findings that multitasking compromised the safety and health of individuals. The study revealed that the majority of respondents had fatigue and stress as the cardinal safety and health risks. With regard to this, Butcher and Rose-Adams (2015) reported that stress has been associated with people who work at more than one job, with fatigue resulting from lack of rest. In this study, stress, which could be physical, social and mental, was associated with factors such as multitasking, travelling long distances, working long hours, lack of sleep, unshared family responsibilities and financial burden. Other health factors associated with stress are burnout, which is a severe psychological strain and physical and emotional exhaustion (Kar & Suar 2014:23–24). However, stress also has the ability to increase individual motivation and can stimulate and enhance an individual’s potential (Sahari et al. 2013:566).

According to Aaronson, Pallikkathayil and Crighton (2013:420), a total of 7 million people reportedly consulted their doctors in a year because of fatigue associated with lack of rest. Fatigue has dire consequences for studying nurses as it leads to higher depression scores, increased risk factors for cardiovascular disease, obesity and poor academic performance (Steege, Pasupathy & Drake 2014:778). Furthermore, fatigue can result in burnout and impaired physical and cognitive functioning, which would inhibit performance in both work and studies (Han, Trinkoff & Geiger-Brown 2014:409).

#### Energy drinks

Another key finding of the study was respondents’ reliance on stimulating drinks or boosters, such as coffee and energy drinks, which contain caffeine. Supporting this, Jovel and Mejia (2017:394) attested that caffeine is the most used psychostimulant in the world. This finding is supported by Turnbull et al. (2017:165), who confirmed that caffeine is a central nervous stimulant found in various plants that are frequently ingested, such as coffee, cocoa beans, tea, guarana berries and kola nuts. Velazquez et al. (2012:167) are of the view that young adults consume energy drinks in an effort to improve their academic performance.

Several authors have outlined the consequences of caffeine consumption. Chaudhary et al. (2016:1193) indicated that caffeine use improves performance in consumers. This was supported by 41% of respondents in this study who indicated that they consumed caffeine to stay alert and 14% who did so to stay awake. The response is supported by Ramamoorthy et al. (2017:898), who confirmed that caffeine use leads to insomnia. Dennison, Rogers and Randolph (2013:468) believed that energy drinks increase stamina, hence six (9%) respondents in this study consumed energy drinks when they felt tired. According to Attila and Çakir (2011:316) and Reid et al. (2014:105), energy drinks improve attention, alertness and reaction time, helping with studies and major projects.

The danger of caffeine consumption is that it predisposes consumers to cardiovascular diseases, such as coronary heart disease, arrhythmia, heart failure, cardiac arrest, stroke and hypertension, palpitations, diuresis, central nervous system stimulation, metabolic acidosis and convulsions (Stacey et al. 2017:S32; Turnbull et al. 2017:169–175). Another danger, as outlined by Jovel and Mejia (2017:394), is hyperexcitability, which can lead to headache.

#### Fast food

A total of 122 (70%) respondents were aware of the health risks of fast food, such as obesity and the associated health problems. According to Rummo et al. (2015:128), fast food is associated with bad eating habits such as binging and higher calorie intake because of their high fat, carbohydrate and sugar content. The high intake of sugar and fats can contribute to obesity. According to Pieroni and Salmasi (2014:95), obesity can lead to death from chronic illnesses such as diabetes, heart disease, stroke and cancer.

#### Financial problems

Some respondents had financial problems because of the additional costs that they had to incur because of their studies. This was particularly so for those individuals who did not have study leave. Despard et al. (2016:8) supported this by saying that ‘study costs even outpace inflation’. Students are usually required to pay substantial amounts for tuition fees, books, travel and accommodation (Despard et al. 2016:8). Respondents in this study indicated that they had to pay their tuition fees, buy study material and travel to their institution of learning from their homes, all of which needed substantial finances. According to the research findings, 31% (55) of the respondents had to travel a distance of more than 150 km to attend their classes, three times a month. For those who could not travel every day, accommodation was sought, usually at great cost. Despard et al. (2016:8–9) found that most students used loans to pay for their education. This led to unmanageable debts, which in turn can contribute to stress (Despard et al. 2016:9).

#### Time management

Most respondents (*n* = 111; 64%) indicated that they experienced problems with time management. To overcome time management problems, students need assistance with how to plan their study schedules and how the schedules can interfere with work and other responsibilities (Owen, Kavanagh & Dollard 2017:508). Pookaiyaudom (2015:268) stated that excellent time management can assist in balancing students’ lifestyle and setting priorities and goals, which will lead to the completion of studies in record time. The consequence of poor time management is stress.

### Safety risks

#### Excessive travelling

Respondents indicated that they had several safety risks associated with travelling. According to Lehrer (2015:224), travelling leads to fatigue and reduces alertness, resulting in poor concentration and accidents.

#### Safety risks associated with shift work

There were 111 (63%) respondents who reported that they worked in shifts. Of these respondents, 69 (62%) indicated that they got very tired from shift work, 16 (14%) experienced insomnia and eight (7%) experienced stress because of their studies. Clendon and Gibbons (2015:1232) stated that nurses working more than 8 h per day are at high risk of accidents and errors, as they are exhausted most of the time. Working night duty can destabilise the family lifestyle and self-care of individuals, as one respondent in Vitale, Varrone-Ganesh and Vu’s (2015:77) reported having a nervous breakdown after being divorced for always being at school or work because of shifts. Shift work and family conflicts contribute to sleep disorders (Zhang, Punnett & Nannini 2017:296). Acutt and Hattingh (2016:173) reported that shift work leads to disturbed sleep, malaise and fatigue, cardiovascular diseases, divorce and substance abuse. Those driving home or to school after a 12-h night shift are prone to accidents as they might be sleepy (Lehrer 2015:224).

## Limitations of the study

The study was contextual in nature, therefore, the results cannot be generalised to all working professional nurses who are studying part-time. The other limitation was that the researcher was unable to follow up on responses where further information was needed as the promotion of anonymity meant that the gathered data could not be linked to respondents.

## Recommendations of the study

It is imperative for people who decide to embark on studying whilst already employed to plan and prepare for this task. These are usually mature people who are already stable and have standing responsibilities. In this instance, studying may be disruptive and demanding physically, emotionally, socially and economically. Employed professional nurses should, therefore, be encouraged to plan their life activities before embarking on any type of study. It is important to get the support of the employer because, invariably, the finance and time aspects are taken care of; seek support from family and sometimes friends for them to award the student status, which will relieve them of some expected responsibilities. They also have to seek support system such as psychologists who can help them to cope with their stress. Employed students should learn time management so that they are able to cope with their multitasking.

## Conclusion

Employed students are an asset to the nation as their intention is, amongst others, to improve their competencies to perform their duties diligently. They also contribute to the country’s economy by ensuring a healthy community. Because of their importance, they need to be psychologically, materially and financially supported during this period, to ensure that they complete their study programmes in good health and ready to plough back the skills gained during the study period.

The study was able to attain its objective of exploring the health and safety issues that affect employed students. The research findings indicated that respondents faced several health risks such as fatigue, stress, cardiovascular disease, stroke, obesity and diabetes, based on activities such as poor eating habits, consumption of caffeine, substance abuse (including alcohol abuse), disrupted sleeping patterns, lack of rest, poor time management and financial burdens.
